# Combining Surveillance Systems: Effective Merging of U.S. Veteran and Military Health Data 

**DOI:** 10.1371/journal.pone.0084077

**Published:** 2013-12-26

**Authors:** Julie A. Pavlin, Howard S. Burkom, Yevgeniy Elbert, Cynthia Lucero-Obusan, Carla A. Winston, Kenneth L. Cox, Gina Oda, Joseph S. Lombardo, Mark Holodniy

**Affiliations:** 1 Armed Forces Health Surveillance Center, US Department of Defense, Silver Spring, Maryland, United States of America; 2 Applied Physics Laboratory, Johns Hopkins University, Laurel, Maryland, United States of America; 3 Office of Public Health, US Department of Veterans Affairs, Washington, District of Columbia, United States of America; 4 US Army Institute of Public Health, US Department of Defense, Aberdeen Proving Ground, Maryland, United States of America; The Australian National University, Australia

## Abstract

**Background:**

The U.S. Department of Veterans Affairs (VA) and Department of Defense (DoD) had more than 18 million healthcare beneficiaries in 2011. Both Departments conduct individual surveillance for disease events and health threats.

**Methods:**

We performed joint and separate analyses of VA and DoD outpatient visit data from October 2006 through September 2010 to demonstrate geographic and demographic coverage, timeliness of influenza epidemic awareness, and impact on spatial cluster detection achieved from a joint VA and DoD biosurveillance platform.

**Results:**

Although VA coverage is greater, DoD visit volume is comparable or greater. Detection of outbreaks was better in DoD data for 58% and 75% of geographic areas surveyed for seasonal and pandemic influenza, respectively, and better in VA data for 34% and 15%. The VA system tended to alert earlier with a typical H3N2 seasonal influenza affecting older patients, and the DoD performed better during the H1N1 pandemic which affected younger patients more than normal influenza seasons. Retrospective analysis of known outbreaks demonstrated clustering evidence found in separate DoD and VA runs, which persisted with combined data sets.

**Conclusion:**

The analyses demonstrate two complementary surveillance systems with evident benefits for the national health picture. Relative timeliness of reporting could be improved in 92% of geographic areas with access to both systems, and more information provided in areas where only one type of facility exists. Combining DoD and VA data enhances geographic cluster detection capability without loss of sensitivity to events isolated in either population and has a manageable effect on customary alert rates.

## Introduction

The availability of accurate and timely information is a critical element needed for an effective response to disease threats, both naturally occurring events such as influenza epidemics and potential terrorist attacks involving biological agents, such as occurred in the United States with the anthrax mailings [1]. Protecting the health and safety of the public requires a well-integrated national biosurveillance enterprise. Among other data sources, biosurveillance includes the collection and analyses of human health indicators such as healthcare utilization. Effective biosurveillance systems leverage robust data collection and analyses performed at the local level, while incorporating a national perspective to provide a broad picture across regions or jurisdictions.

In 2011, the Department of Veterans Affairs (VA) system had 8.6 million enrolled Veteran patients and 79.8 million outpatient visits [2] and the Department of Defense (DoD) Military Health System had 9.7 million eligible beneficiaries and 38 million outpatient visits [3]. The Departments are among the largest healthcare systems providing care in the United States. The Departments use distinct versions of a system known as the Electronic Surveillance System for the Early Notification of Community-Based Epidemics (ESSENCE) and also report surveillance data to BioSense, a national biosurveillance platform provided by the Centers for Disease Control and Prevention (CDC) and administered by the Association of State and Territorial Health Officers [4]. However, DoD and VA data are evaluated separately for threats to health, and data suitable for population-based health surveillance are not routinely shared between these agencies. 

Recently, a retrospective analysis combining data from VA and DoD facilities in North Chicago, IL, was used to examine seasonal influenza trends, a gastrointestinal (GI) illness epidemic and a heat-related event. This provided the first example of the possible benefits of performing joint VA and DoD biosurveillance [5]. Then, in July of 2012, the White House released the first-ever National Strategy for Biosurveillance where President Barack Obama called for “a coordinated approach that brings together Federal, State, local, and tribal governments…” [6]. The strategy proposes “to integrate and enhance national biosurveillance efforts,” and outlines four focus areas as enablers for strengthening biosurveillance. These include: integrate biosurveillance capabilities, such as through information sharing arrangements and across traditional organizational lines; build capacity across our distributed national biosurveillance architectures; foster innovation such as combining information to project what is likely to transpire; and strengthen partnerships through the sharing of information between and among Federal, State, local, tribal, territorial, private, nongovernmental, academic and other participants [6]. 

Based on this recognized need, we submitted a proposal to the Joint Incentive Fund, a program designed to facilitate the coordination, use, or exchange of health care resources, with the goal of improving access to, and quality and cost effectiveness of, the health care provided to beneficiaries of both the DoD and VA [7]. Our aim was to determine if we could improve early detection and situational awareness of health events of regional and national significance through a consolidated system that maximizes both the sample size and diversity of the populations monitored and reflects the spirit and goals of improving integration and coordination as outlined in the National Biosurveillance Strategy. Here we describe the geographic coverage and outpatient visit characteristics of the two populations, the relative timeliness in detection of the two systems for seasonal influenza epidemics by region for both separate and combined data, and the effect of combining systems on spatial cluster detection. We demonstrate the utility of a joint DoD and VA public health surveillance system that can inform not only the two Departments, but the nation’s public health awareness.

## Materials and Methods

### Data

Electronic International Classification of Diseases, 9^th^ Revision (ICD-9) codes from outpatient medical visits from all DoD and VA facilities for October 1, 2006, to September 30, 2010 (Fiscal Years 2007–2010) were available for analysis from the Departments’ electronic health records. The dataset included over 137.7 million DoD visits from 131 hospitals and ambulatory care centers which include 362 separate clinics and over 253 million VA visits from 128 hospitals and 786 clinics.

### Coverage determination

We used the US Office of Management and Budget’s core-based statistical area (CBSA) to group healthcare data from the respective VA and DoD systems by megapolitan (>1 million population), metropolitan (50,000-1 million population) and micropolitan (10,000-50,000 population) areas. We performed frequency analyses and mapped coverage of the VA and DoD medical systems in these CBSAs by number of hospitals, number of clinics, counts of all visits, and counts by age for 0-17, 18-44, 45-64 and over 65 years.

### Comparability of systems for temporal detection

To assess temporal detection patterns in VA and DoD data, we retrospectively evaluated two influenza epidemics that were expected to affect covered populations in many CBSAs. The first event was seasonal influenza dominated by an H3N2 strain that began in December 2007 and continued until early April 2008. This event was more severe than other seasonal outbreaks since 2005, especially among the elderly. The second event was the fall wave of the novel H1N1 influenza pandemic in 2009. The fall wave was classified similarly as moderately severe by the CDC [8]; however, the burden of morbidity was unusually high among children and young adults [9]. 

We included the following clinic types for analysis: internal medicine, pediatrics, adolescent medicine, family practice, primary care, urgent care, geriatric clinics, women’s primary care, infectious diseases and emergency medicine. We grouped visits into an influenza-like-illness (ILI) syndrome category if the assigned ICD-9 code(s) for the visit matched a defined ICD-9 code for the ILI syndrome group. The ICD-9 codes used to determine inclusion in the ILI syndrome group in ESSENCE were originally selected from a study correlating ICD-9 codes with positive influenza laboratory tests [10]. To choose an ICD-9 set for this current analysis, we analyzed code frequencies in each Department’s system to determine relative frequency and comparability and chose a common ILI classification (Table S1 in File S1). 

We formed weekly CBSA counts by summing the ILI-related visit counts from all facilities within the CBSA. We applied ESSENCE alerting algorithms [11] to weekly CBSA-level outpatient data and analyzed the two data streams (DoD and VA) separately. We expressed timeliness in weeks rather than days to reduce the effects of reporting or system acquisition delays. We applied the default ESSENCE alerting algorithm [11] to all time series of weekly ILI counts for all CBSAs with both VA and DoD healthcare facilities for the four years. For meaningful timeliness comparison, we restricted the analysis to the 93 CBSAs with at least two ILI-related visits per week in both DoD and VA datasets, primarily megapolitan and metropolitan areas. We chose November 1, 2007, to March 31, 2008 (2007–2008 seasonal influenza), and September 1, 2009, to December 31, 2009 (2009 novel H1N1 pandemic fall wave) as representative moderately severe influenza outbreaks [8]. In brief, for derived time series with systematic or cyclic behavior, the ESSENCE algorithm defaults to regression models including seasonal and day of week effects to calculate expected counts, and produces alerts when visit counts are statistically higher than expected. For sparser time series, an adaptive control chart is the default. For both influenza epidemics, we compared the date of the first alert occurring at the p<0.01 statistical significance level and the number of significant alerts for each CBSA in each of the DoD and VA outpatient datasets. 

### Comparability of systems for cluster detection

To analyze VA and DoD surveillance data for potential disease outbreaks in space and time, we calculated spatial scan statistics using software introduced in [12] and identified statistically significant clusters of events. Outpatient visit records from both systems contain a patient zip code field that is completed in over 94% of records. Using records grouped using the patient zip code field, we estimated visit distributions for both DoD and VA datasets and applied a published spatial scan statistics implementation [10] to the separate and combined datasets. 

Input data files in the combined and separate VA and DoD analyses were matrices of daily ILI or gastrointestinal (GI) syndrome visit counts with a common ICD-9 code grouping chosen for both VA and DoD systems (Tables S1-S2 in File S1). GI syndrome codes were standard codes historically used by DoD and VA ESSENCE. Table S2 in File S1 provides the GI codes and indicates the top 10 that we used in the medical records in these analyses. Matrix rows were consecutive days, columns were patient residence zip codes, and entry (i,j) was the number of visits on day i from zip code j. We made these files for DoD data, VA data, and combined data.

To assess the number of significant clusters determined from combining datasets, we used data from three separate regions – Baltimore/Washington, DC (dominated by DoD data), Los Angeles, CA (mainly VA data) and Tampa, FL (both represented). For each region, we ran sets of 1,672 consecutive single-day analyses for cluster determination trials from October 1, 2006 through September 30, 2010 for ILI and GI syndrome data. To examine background alerting rates in these sets, we tabulated counts of clusters with p-values no larger than 0.001 using the standard rank-based p-value introduced in [13] for spatial scan statistics. 

In addition to examining the alert rate, we performed focused runs to detect known events without advanced knowledge regarding what clustering to expect for outbreaks in New York (GI, January-March 2010), California (ILI, December 2007-April 2008 and September-December 2009), and New Jersey (GI, January-March 2010). We did not receive prior reports of any spatial clustering in these outbreaks. 

## Results

### Coverage determination

**Figure 1:**
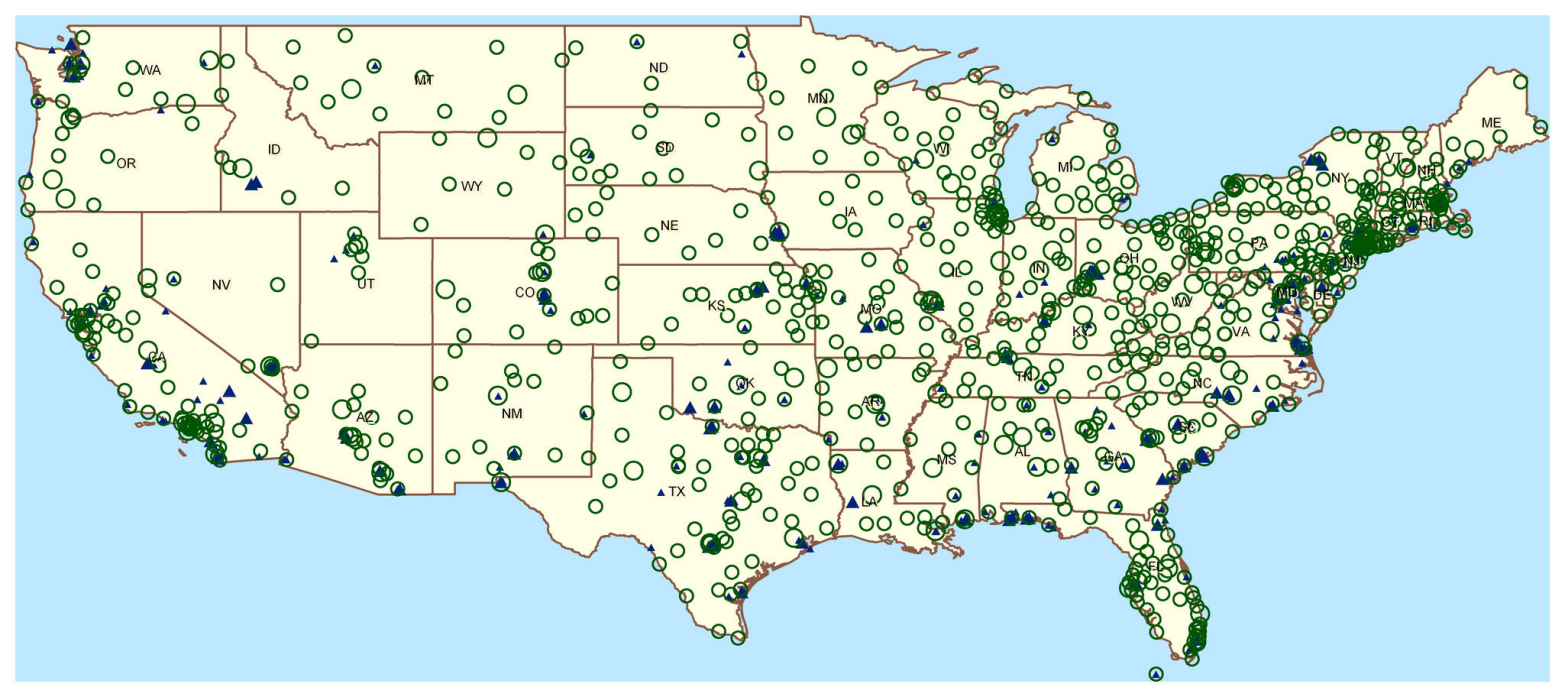
Department of Defense and Veterans Affairs medical facilities in the continental United States. Locations of VA hospitals (large green circles) and clinics (small green circles) and DoD hospitals (large blue triangles) and clinics (small blue triangles) in the continental US.

We identified a total of 939 CBSAs, with generally diffuse geographic coverage by VA facilities and higher concentration in larger metro and mega areas for DoD facilities (Figure 1). Of the 51 mega CBSAs, all had at least one VA facility and 63% had a DoD facility (Table S3 in File S1). Coverage was sparser for the metro CBSAs and lighter still for the micro CBSAs. While the VA coverage was greater in terms of total number of visits for all CBSA levels (253 million vs. 137 million), the DoD visit volume exceeded the VA visit volume in 67 of the 132 CBSAs covered by facilities in both systems (Table S3 in File S1) . Patient age distribution differed sharply, with >85% of the VA patients being over 45 years of age compared to 22% of DoD patients. For all CBSAs, the overall VA/DoD visit ratio was 1.92, but the ratio for 0-17 years was 0.004, 18-44 years 0.33, 45-64 years 5.20 and >65 years 11.63. 

### Comparability of systems for temporal detection

**Figure 2:**
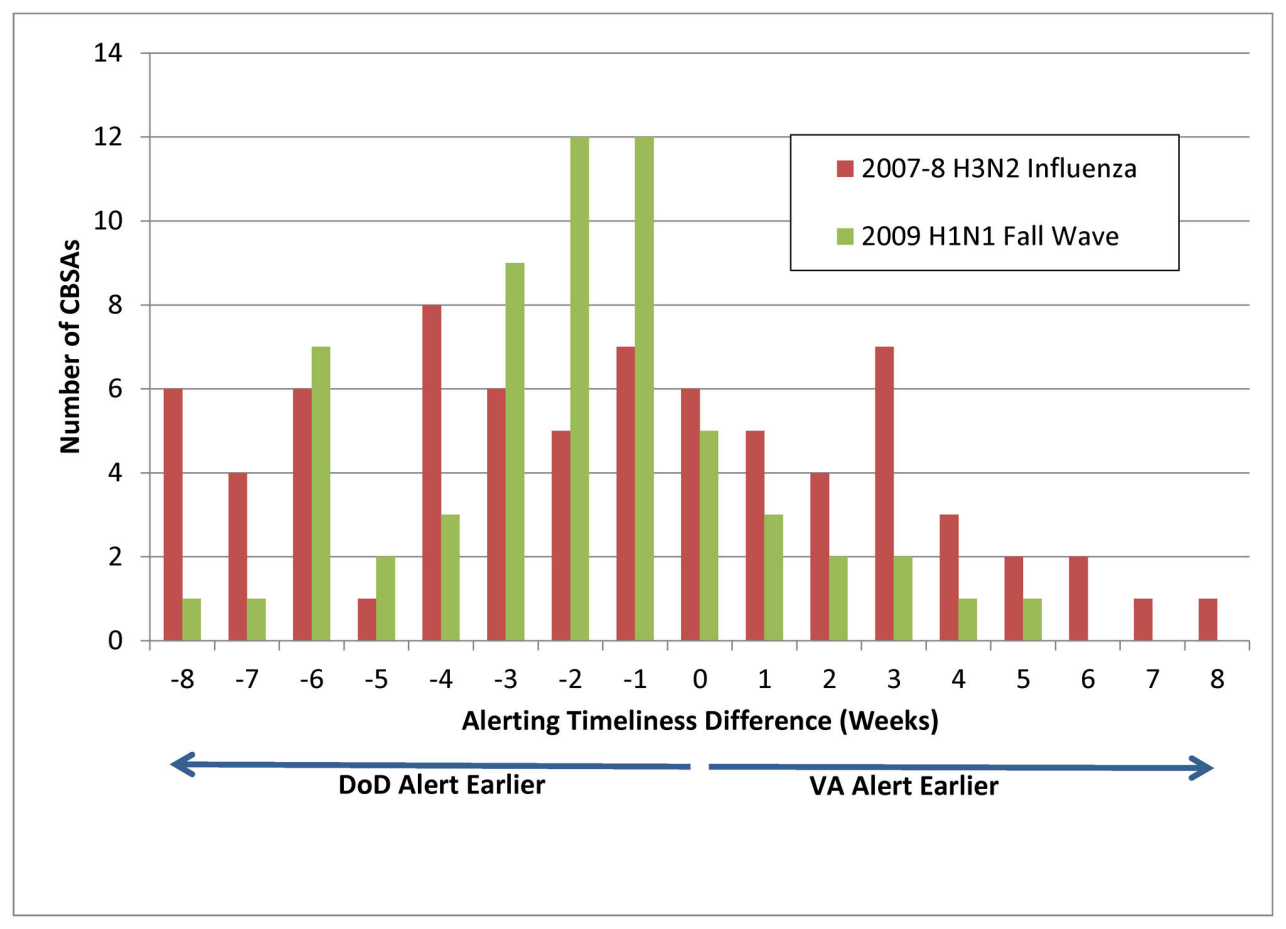
Delay in weeks between Department of Defense and Veterans Affairs alerting for two influenza seasons. The height of each bar is the number of Core-Based Statistical Areas (CBSAs). The x-axis is delay lengths in weeks, with the x-axis origin indicating no alerting delay between systems, negative delays indicating earlier DoD alerting and positive delays for earlier VA alerting. Red bars show relative timeliness for the 2007-8 H3N2 Influenza epidemic, green bars show timeliness for the 2009 novel H1N1 fall pandemic.

Tables 1 and 2 summarize the timeliness comparison for ILI in alert weeks. Based on the curve for timeliness of detection (Figure 2), we removed CBSAs from the analysis if they were outliers with more than eight weeks between the DoD and the VA alerting. Therefore, we report 77 and 79 of the CBSAs for the 2007-2008 season influenza outbreak and 2009 pandemic, respectively. We detected seasonal 2007-2008 influenza at significant alert levels in at least one of the systems in all CBSAs, and detected the fall 2009 pandemic influenza wave in 76 of 79 included in the analysis (96.2%). Detection occurred in the same week for both VA and DoD in only six CBSAs (7.8%) for the seasonal event and in only five CBSAs (6.3%) for the pandemic (Figure 2). When we found a timeliness difference, detection in the DoD data tended to occur earlier, but not uniformly so (Table 1). Considering the 2007-2008 season for all analyzed CBSAs, detection occurred earlier or only in DoD data for 45 CBSAs (63.4%) and in VA data for 26 CBSAs (36.6%, significantly different at the 95% confidence level). For the pandemic influenza year that affected primarily younger patients, DoD data detected earlier or only for 59 CBSAs (74.7%), but 12 CBSAs (15.2%) still detected earlier or only in VA data, and for this event the earlier DoD detections occurred in all CBSA sizes. Restriction to CBSAs with higher patient loads (more than 10 ILI visits/week) gave similar results (Table 2). Overall, DoD tended to alert earlier across all influenza seasons examined; however, VA data alerted earlier in a greater number of CBSAs during the typical H3N2 seasonal flu when compared to the 2009 H1N1 influenza pandemic. 

**Table 1:** Alerting timeliness comparison of Department of Defense (DoD) and Veterans Affairs (VA) outpatient records associated with influenza-like illness (ILI) during two different influenza events and both events combined for Core-Based Statistical Areas (CBSAs) that have both DoD and VA facilities and have at least 2 ILI patients/week stratified by alerting timeliness.

Timeliness (weeks)	Seasonal Influenza 2007-08 H3N2	Pandemic 2009 Fall H1N1	Both Influenza Events
Alert Same Week	6	5	11
DoD Alerts Earlier	43	47	90
VA Alerts Earlier	25	9	34
Only DoD Alerts	2	12	14
Only VA Alerts	1	3	4
Neither Alerts	0	3	3
Total	77	79	156

**Table 2:** Alerting timeliness comparison of Department of Defense (DoD) and Veterans Affairs (VA) outpatient records associated with influenza-like illness (ILI) during two different influenza events and both events combined for Core-Based Statistical Areas (CBSAs) that have both DoD and VA facilities and have at least 10 ILI patients/week stratified by alerting timeliness.

Timeliness (weeks)	Seasonal Influenza 2007-08 H3N2	Pandemic 2009 Fall H1N1	Both Influenza Events
Alert Same Week	3	5	8
DoD Alerts Earlier	25	36	61
VA Alerts Earlier	15	6	21
Only DoD Alerts	0	1	1
Only VA Alerts	0	1	1
Neither Alerts	0	2	2
Total	43	51	94

To describe delay lengths, defined as alerting timeliness difference between DoD and VA data, Figure 2 presents the distribution of week alerted in DoD data minus week alerted in VA data for the two selected outbreaks. For the H3N2 seasonal influenza, eight CBSAs alerted four weeks earlier in DoD data than VA data but for the H1N1 fall wave, only three CBSAs alerted with the same four week advantage to the DoD data. Conversely, seven CBSAs alerted three weeks earlier in VA data for the H3N2 epidemic but only two such CBSAs for the H1N1 pandemic. The fact that both bar plots are skewed to the left shows a general timeliness advantage to the DoD data. The delays for VA data occurred one to four weeks for most CBSAs with some longer delays. Again, the timeliness advantage was not uniform; for the 2007-2008 influenza season, a one to four week timeliness advantage occurred in the VA for a total of 19 CBSAs. For the pandemic influenza 2009 effect, whose burden was mainly among young adults, the delays are more clearly skewed towards earlier alerting within the DoD system.

### Comparison of cluster detection analyses for combined and separate datasets

To investigate cluster determination rates in several environments, we determined over 45% of the zip codes are represented in both systems (Table 3). Counts of derived clusters statistically significant for p-values below 0.001 are given in Table 4. In all three regions, In Los Angeles, the remaining zip codes are dominated by VA data, in the Baltimore/DC area, they are dominated by DoD data, with a relative even distribution in Tampa (Table 3). The amount of clustering varies directly with the total number of zip codes, with ILI clusters outnumbering GI clusters in each run scenario that included more than 10 zip codes. Table 4 demonstrates that combining datasets maintained a relatively stable alert burden. 

**Table 3:** Number of zip codes represented by metropolitan area for case clusters combining Department of Defense (DoD) and Veterans Affairs (VA) datasets from October 1, 2006 - September 30, 2010.

	Number of Zip Codes with DoD Only	Number of Zip Codes with VA Only	Number of Zip Codes with Both VA and DoD
Los Angeles	6	314	369
Baltimore/DC	495	70	534
Tampa	36	128	226

**Table 4:** Number of alerts using scan statistics to find significant case clusters (p<0.001)* by metropolitan area and syndrome group combining Department of Defense (DoD) and Veterans Affairs (VA) datasets from October 1, 2006 - September 30, 2010.

	Number with Joint Data Clusters	Number with DoD-only Clusters	Number with VA- only Clusters
Los Angeles, ILI	24	5	26
Los Angeles, GI	7	10	9
Baltimore/DC, ILI	142	148	17
Baltimore/DC, GI	52	49	11
Tampa, ILI	15	5	13
Tampa, GI	2	4	4

^*^ Influenza-like illness (ILI) and gastrointestinal illness (GI) visits are the outcome variables aggregated by patient zip code. The p-values were computed using the rank-based method introduced in [11] as p-value = (rank of the maximum cluster statistic) / (1 + number of trial runs).

### Seeking clusters retrospectively from data during intervals of known outbreaks

For the focused cluster determination runs, Table 5 shows the amount of clustering during the reported event and also in the ambient data. We chose the first scenario based on two reports from New York State – a norovirus outbreak at a DoD clinic at West Point from the end of January 2010 lasting several weeks, and reports of excess viral gastrointestinal cases across the state during approximately the same weeks reported from VA clinics. For the West Point event, we found clusters significant at p<0.01 beginning on January 26, a few days before the reported event started, and in runs of seven of the next ten days, from zip codes near West Point. We found these clusters in the DoD-only and the combined data runs, but not in the VA-only data. We did not find any clustering evidence corresponding to the VA GI reports or linking any VA events to the West Point clusters. However, the combined data results included three days of GI clustering in overlapping groups of 3-8 patient zip codes on October 26-28, 2009. These clusters included a total of 68 visits, and we found no significant clustering in these zip codes in the separate DoD or VA runs.

**Table 5:** Results of sets of cluster determination daily runs conducted from August 9, 2009 to June 10, 2010 using data from regions of reported outbreak events.

	New York	California	New Jersey
Syndrome	GI	ILI	GI
Number of VA Only Zip Codes	1,392	203	487
Number of VA Clusters, p<0.01	6	6	13
Median Number of VA Cases	2,492	1,044	704
Number of DoD Only Zip Codes	252	288	199
Number of DoD Clusters, p<0.01	24	149	6
Median Number of DoD Cases	1,094	17,156	280
Number of Combined Zip Codes	1,487	289	559
Number of Combined Clusters, p<0.01	26	138	16
Median Number of Combined Cases	3,500	17,972	980

We chose the second scenario based on another reported DoD GI event at Fort Dix, New Jersey with cases reported from early January 2010, increasing through mid-March. Early in this interval, results from this scenario yielded intermittent outbreak clusters in the combined and DoD datasets, but not until the caseload began to increase in February. We found clusters of over seven cases on four consecutive days in the first week of March. The VA data indicated twice the GI visit counts of the DoD data, but without clustering to corroborate the Ft. Dix event.

We chose the third scenario as the more geographically widespread fall wave of the novel H1N1 pandemic of 2009, starting in San Diego, California. In those runs, the large, heterogeneous group of care facilities yielded large, highly significant clusters, but day-to-day cluster evolution could not be observed. The runs using combined data were dominated by the DoD volume in the San Diego region, as seen in Table 5. Runs using the VA data alone did not produce significant clustering. 

## Discussion

The coverage analysis presented provides a picture of two complementary biosurveillance systems with evident benefits to the national health picture for information fusion. Based on analysis of outpatient visits covering a 4-year period, the overall VA system patient volume roughly doubles that of the DoD system. Geographically, the location of VA care facilities mirrors the spread of former Service members throughout the US while DoD facilities are mainly clustered around larger military installations, many of which are near regions of strategic importance. Observers with access to both DoD and VA data would get sharply different impressions of the systems’ relative population coverage depending on the region. In much of the geographic area of the U.S. away from military bases, most data are from VA hospitals and clinics. However, in some of the most densely populated metropolitan regions, the majority of data are from DoD hospitals and nearby clinics. 

Roughly 80% of VA visits are for patients at least 45 years old, and, corresponding to limited family benefits, the coverage of patients under 17 is relatively negligible. The DoD facility patient base shows over 75% of visits for patients below age 45 and a substantial 10-15% of visits for patients below active duty age who are covered as children of Service members or above active age as retirees. This complementarity supports the fusion of information from DoD and VA healthcare systems, with the caveat that even an effectively combined system might not be useful for tracking illness among younger patients away from population centers and military bases. Given the importance of age as a risk factor for many health threats, an epidemiologist from the VA or DoD would want access to both systems for a comprehensive population health status. State and local health departments who participate in BioSense should soon be able to see some DoD and VA surveillance data, but they also would benefit from access to a combined system when information about their regions is available. 

The temporal analysis provides evidence of a potential detection advantage in either system depending on where an outbreak occurs and on the population affected. The general timeliness advantage for the DoD outpatient data relative to VA data is consistent with the age distribution and the fact that influenza epidemics, for example, often appear first and spread quickly among the young. The sharper timeliness differences for the H1N1 pandemic particularly illustrate this age effect. However, for more common seasonal influenza outbreaks, the DoD timeliness advantage is less dramatic, with earlier VA alerting in over a third of CBSAs. Furthermore, the DoD timeliness is balanced by the fact that many metropolitan and most micropolitan CBSAs are served by VA but not DoD facilities. Another finding supporting the importance of combining data from the VA and DoD surveillance systems is that same-week detection occurred in only six out of 93 CBSAs covered substantially by both systems for the 2007-8 influenza epidemic, and only in five CBSAs for the 2009 H1N1 fall wave. 

One limitation of the study is the unavailability of CBSA-specific outbreak dates. As the dates of the influenza outbreaks could vary widely, even for neighboring CBSAs because of differences in introduction of the strains caused by travel and commuting among affected populations, we did not attempt this analysis. Therefore, we cannot be certain that we captured the influenza outbreaks correctly for comparison. In addition, during analysis of timeliness comparisons, some CBSAs had alerting differences of up to 20 weeks. Due to the inability to accurately determine in retrospect whether this was truly a difference in detection or comparing two distinct outbreaks, we limited the analysis to those with detections in both systems within an eight week range. The eight week window could be too conservative or liberal, and we could be under or over-estimating the differences between detection in the VA and DoD.

The cluster detection analysis had additional challenges. Calculations for the ILI syndrome in the DoD and combined datasets yielded many statistically significant clusters, more than one per week in data-rich regions with multiple, heterogeneous facilities such as the San Diego scenario in Table 5. In such scenarios, the mobile active-duty DoD population at some installations may have resulted in unrepresentative historical baselines. Accurate denominator data for such installations cannot be current for several reasons, including mission security. Another limitation is that the available outbreak information was only for large events, usually with over 50 cases but with no details regarding the distribution of patient residences. Nevertheless, the cluster detection analysis produced three important findings: 1) Known outbreaks that produced clusters in the DoD or VA systems were not masked when the datasets were combined; 2) the number of significant clusters increased little or not at all in the combined datasets; and 3) merging the data did produce significant clusters that were not detected using either DoD or VA data alone. For events examined in this analysis, clustering itself yielded an occasional but not consistent timeliness advantage. More detailed outbreak information is needed to quantify the timeliness and sensitivity advantages of combining datasets. In view of the varied distribution of DoD and VA data across the U.S., Figure 1 and the anecdotal examples above suggest that there are many geographic regions where a health monitor in one of the systems would benefit from analysis of combined data.

Another limitation of this study is the retrospective nature of the data and the consequent inability to test the performance of a dual system to provide accurate situational awareness during an actual health event in real time. In addition, the use of ICD-9 codes for surveillance can provide inaccurate data secondary to miscodings. The separate DoD and VA systems currently analyze additional data sources such as chief complaints, laboratory test orders and results and pharmacy orders which also can provide incorrect information, but assists in alert verification. In the future, the merger of these and future (e.g., inpatient) data sources could improve the robustness and accuracy of system performance.

In addition, with a combined system, the question arises whether modifications to the default ESSENCE alerting algorithms would be needed considering the different population demographics, geography and historical disease baselines. This may be needed, but more with regard to regional details such as DoD/VA data composition, facility distribution and regional surveillance concerns and resources, than to detailed population modeling. For example, the ILI-specific alert rates at some large metro areas were high compared to other syndromes and other areas. The underlying reasons for these differences (e.g., different facility types with more referral and acute care or more higher-risk elderly at some) should be explored and a decision made either that the elevated alerting is both justifiable and manageable given the local investigation resources, or the thresholds need to be altered. In summary, given the complex and evolving combined data picture, we would not immediately modify algorithm settings based purely on statistical considerations, but instead supply auxiliary information or visualization to facilitate interpretation based on the end user understanding of the local situation.

Currently, through the JIF funding, we have demonstrated successful data exchange between the VA and DoD and created a joint ESSENCE application which contains approximately two years of historic surveillance data from all facilities from both agencies for evaluation. We are planning a cost-benefits analysis to study the merits of developing, converging to, and maintaining a single ESSENCE system that would be used by both agencies. We hope to establish real-time data feeds from both agencies into a single application, consolidate our existing system into a unified platform which will eliminate the need to maintain separate ESSENCE programs and eventually move this unified application into a secure cloud environment. When we accomplish this, we will have taken a big step in the right direction toward a true linkage of federal agency biosurveillance capabilities and health data exchange.

## Supporting Information

File S1 **This file contains Table S1-Table S3.** Table **S1**, Diagnosis code classification for influenza-like-illness syndrome group analysis. Table **S2**, Diagnosis code classification for gastrointestinal syndrome group analysis. Table **S3**, Counts of Core-Based Statistical Areas (CBSAs) for Veterans Affairs (VA) and Department of Defense (DoD) medical facilities for three population scales. A. Distribution of CBSAs with VA and DoD facilities by population density. B. Comparison of number of patient visits between VA and DoD in each of the population scales by CBSAs which have both systems.
(DOCX)
