# Supplementary material for: Combining Surveillance Systems: Effective Merging of U.S. Veteran and Military Health Data
Source: PLoS One. 2013 Dec 26;8(12):e84077. doi: 10.1371/journal.pone.0084077 (PMC3873400; doi:10.1371/journal.pone.0084077)
Supplement: File S1 — This file contains Table S1-Table S3. Table S1, Diagnosis code classification for influenza-like-illness syndrome group analysis. Table S2, Diagnosis code classification for gastrointestinal syndrome group analysis. Table S3, Counts of Core-Based Statistical Areas (CBSAs) for Veterans Affairs (VA) and Department of Defense (DoD) medical facilities for three population scales. A. Distribution of CBSAs with VA and DoD facilities by population density. B. Comparison of number of patient visits between VA and DoD in each of the population scales by CBSAs which have both systems. (DOCX) [file pone.0084077.s001.docx]

**Supplemental Data**

**Table S1.**

| ICD-9 Code(s) | Description |
| --- | --- |
| 780.60 and 784.1 | Fever and Sore Throat |
| 780.60 and 786.2 | Fever and Cough |
| 780.60 and 382.9 | Fever and Acute Otitis Media |
| 780.60 and 461.9 | Fever and Sinusitis NOS |
| 460 | Acute Nasopharyngitis |
| 462 | Acute Pharyngitis |
| 465.9 | Upper Respiratory Infection |
| 466.0 | Acute Bronchitis |
| 486 | Pneumonia NOS |
| 490 | Bronchitis NOS |

**Table S2.**

| ICD-9 Code | Description |
| --- | --- |
| 001.0 | Cholera due to *Vibrio cholera* |
| 001.9 | Cholera unspecified |
| 003.0 | Salmonella gastroenteritis |
| 003.8 | Other specified *Salmonella* infections |
| 003.9 | Salmonella infection |
| 004.0 | *Shigella dysenteriae* |
| 004.1 | *Shigella flexneri* |
| 004.2 | *Shigella boydii* |
| 004.3 | *Shigella sonnei* |
| 004.8 | Other specified *Shigella* infections |
| 004.9 | Shigellosis, unspecified |
| 005.0 | Staphylococcal food poisoning |
| 005.2 | *Clostridium perfringens* food poisoning |
| 005.3 | Food poisoning due to other *Clostridia* |
| 005.4 | *Vibrio parahaemolyticus* food poisoning |
| 005.81 | *Vibrio vulnificus* food poisoning |
| 005.89 | Other bacterial food poisoning |
| 005.9 | Food poisoning |
| 008.00 | Intestinal infection due to *E. coli* |
| 008.01 | Intestinal infection due to enteropathogenic *E. coli* |
| 008.02 | Intestinal infection due to enterotoxigenic *E. coli* |
| 008.03 | Intestinal infection due to enteroinvasive *E. coli* |
| 008.04 | Intestinal infection due to enterohemorrhagic *E. coli* |
| 008.09 | Intestinal infection due to other *E. coli* infections |
| 008.2 | Intestinal infection due to *Aerobacter aerogenes* |
| 008.3 | Intestinal infection due to *Proteus* |
| 008.41 | Intestinal infection due to *Staphylococcus* |
| 008.43 | Intestinal infection due to *Campylobacter* |
| 008.44 | Intestinal infection due to *Yersinia enterocolitica* |
| 008.45* | Intestinal infection due to *Clostridium difficile* |
| 008.46 | Intestinal infections due to anaerobes |
| 008.47 | Intestinal infection due to other gram-negative bacteria |
| 008.49 | Intestinal infection due to other organisms |
| 008.5 | Bacterial enteritis |
| 008.61 | Enteritis due to rotavirus |
| 008.62 | Enteritis due to adenovirus |
| 008.63 | Enteritis due to Norwalk virus |
| 008.64 | Enteritis due to other small round viruses |
| 008.67 | Enteritis due to *Enterovirus* |
| 008.69 | Viral enteritis |
| 008.8 | Intestinal infection, other organism |
| 009.0 | Infectious colitis, enteritis or gastroenteritis |
| 009.1 | Colitis, enteritis, gastroenteritis presumed infectious |
| 009.2 | Infectious diarrhea |
| 009.3 | Diarrhea presumed infectious |
| 078.82 | Epidemic vomiting syndrome |
| 535.00 | Acute gastritis |
| 535.01 | Acute gastritis with hemorrhage |
| 535.40* | Other specified gastritis |
| 535.41 | Other gastritis with hemorrhage |
| 535.50* | Unspecified gastritis and gastroduodenitis |
| 535.51 | Unspecified gastritis and gastroduodenitis with hemorrhage |
| 535.60* | Duodenitis |
| 535.61 | Duodenitis with hemorrhage |
| 536.2 | Persistent vomiting |
| 555.0 | Regional enteritis, small intestine |
| 555.1 | Regional enteritis, large intestine |
| 555.2 | Regional enteritis of small intestine with large intestine |
| 558.2 | Toxic gastroenteritis |
| 558.9* | Unspecified noninfectious gastroenteritis and colitis |
| 569.9 | Unspecified disorder of intestine |
| 787.01* | Nausea with vomiting |
| 787.02* | Nausea |
| 787.03* | Vomiting |
| 787.3* | Flatulence, eructation and gas pain |
| 787.91* | Diarrhea |

*In the top ten for usage in medical records analyzed**.**

**Table S3.**

**A.**

| Coverage Summary | Mega CBSAs (Population > 1 million) | Metro CBSAs (Population 50,000 – 1 million) | Micro CBSAs (Population 10,000-50,000) |
| --- | --- | --- | --- |
| Both systems | 32 | 94 | 6 |
| Neither system | 0 | 149 | 244 |
| DoD only | 0 | 16 | 5 |
| VA only | 19 | 292 | 82 |

**B.**

| Visit Counts | Mega CBSAs | Metro CBSAs | Micro CBSAs |
| --- | --- | --- | --- |
| DoD > VA | 6 | 56 | 5 |
| DoD < VA < 5xDoD | 10 | 24 | 1 |
| VA > 5xDoD | 16 | 14 | 0 |
